# Temporal Bone Squamous Cell Carcinoma: Molecular Markers Involved in Carcinogenesis, Behavior, and Prognosis: A Systematic Review

**DOI:** 10.3390/ijms23094536

**Published:** 2022-04-20

**Authors:** Lara Alessandrini, Laura Astolfi, Leonardo Franz, Erica Gentilin, Antonio Mazzoni, Elisabetta Zanoletti, Gino Marioni

**Affiliations:** 1 Department of Medicine-DIMED, University of Padova, 35128 Padova, Italy; lara.alessandrini@aopd.veneto.it; 2 Bioacoustics Research Laboratory, Department of Neuroscience-DNS, University of Padova, 35129 Padova, Italy; laura.astolfi@unipd.it (L.A.); erica.gentilin@unipd.it (E.G.); 3 I–APPROVE—International Auditory Processing Project in Venice, Department of Neurosciences, University of Padova, Santi Giovanni e Paolo Hospital, ULSS3 Serenissima, 30122 Venezia, Italy; 4 Department of Neuroscience-DNS, Otolaryngology Section, University of Padova, 35128 Padova, Italy; antonio.mazzoni@libero.it (A.M.); elisabetta.zanoletti@unipd.it (E.Z.); 5 Guided Therapeutics (GTx) International Scholarship Program, University Health Network, Toronto, ON M5G 1L7, Canada

**Keywords:** temporal bone, squamous cell carcinoma, prognosis, biological markers, target therapy

## Abstract

Temporal bone squamous cell carcinoma (TBSCC) is an uncommon malignancy with a poor prognosis in advanced cases. The dismal outcome of advanced TBSSC cases is largely due to the cancer’s local aggressiveness and the complex anatomy of this region, as well as to persistent pitfalls in diagnosis and treatment. Molecular changes occur in malignancies before any morphological changes become visible, and are responsible for the disease’s clinical behavior. The main purpose of this critical systematic review is to assess the level of knowledge on the molecular markers involved in the biology, behavior, and prognosis of TBSCC. A search (updated to March 2022) was run in PubMed, Scopus, and Web of Science electronic databases without publication date limits for studies investigating molecular markers in cohorts of patients with primary TBSCC. The search terms used were: “temporal bone” OR “external auditory canal” OR “ear”, AND “cancer” OR “carcinoma” OR “malignancy”. We preliminarily decided not to consider series with less than five cases. Twenty-four case series of TBSCC were found in which different analytical techniques had been used to study the role of several biomarkers. In conclusion, only very limited information on the prognostic role of molecular markers in TBSCC are currently available; prospective, multi-institutional, international prognostic studies should be planned to identify the molecular markers involved in the clinical behavior and prognosis of TBSCC. A further, more ambitious goal would be to find targets for therapeutic agents able to improve disease-specific survival in patients with advanced TBSCC.

## 1. Introduction

Temporal bone squamous cell carcinoma (TBSCC) is extremely uncommon, accounting for <0.2% of head and neck squamous cell carcinomas (SCCs) [1]. More frequently, these SCCs originate from the external auditory canal (EAC) and extend into the temporal bone [2]. A 2019 literature review of TBSCC by Lovin and Gidley [3] revealed that the 5-year disease-free survival (DFS) and disease-specific survival (DSS) rates for combined T1 and T2 tumors ranged from 67% to 100% and from 92% to 100%, respectively; and the 5-year DFS and DSS rates for combined T3 and T4 tumors ranged from 41% to 59% and from 48% to 65%, respectively. The dismal outcome of advanced TBSSC cases is largely due to the cancer’s local aggressiveness and the complex anatomy of this region, as well as to persistent pitfalls [2] in the diagnosis and treatment of the tumor. Reliable clinical and pathological prognostic factors are still lacking [4]. This is mainly due to: (i) the small number of cases reported in all available series; (ii) the histological heterogeneity of several case series (different histotypes showing different tumor behavior, local invasiveness, and metastatic pathways); (iii) the lack of universal acceptance on a staging system (making it difficult to compare patient outcomes and the efficacy of different treatments); (iv) inadequacy of the current imaging methods to assess in detail the tumor extent, both at primary and secondary sites; (v) the non-standardized reporting on the pathology-based surgical margins, which is an unsolved issue, particularly when the tumor invades bone [5,6]. The last aspects are the reasons for the current defective assessment of malignancy extent and insufficient knowledge of the modalities of tumor growth in the temporal bone. As for therapy, several issues are still debated, including the choice between en bloc and piecemeal primary surgery for TBSCC removal [7], and the role of elective neck dissection [8]. Although radiotherapy seems to be an effective adjuvant therapy in advanced cases, its role in low-stage tumors, and as a primary treatment, has yet to be established. The value of chemotherapy is also still unclear [1].

Molecular changes occur in malignancies before any morphological changes become visible, and are responsible for the disease’s clinical behavior. A biomarker has been defined as “any substance, structure, or process that can be measured in the body or its products and influence or predict the incidence of outcome or disease” [9]. In clinical practice, the ideal biomarker should have: (i) prognostic value; (ii) a significant capacity to predict the efficacy of specific treatments; and (iii) the features needed for it to become the target of integrated therapeutic approaches. Identifying biomarkers that reflect the biological features of TBSCC may be crucial in promoting the development of more effective integrated therapeutic strategies to improve DSS for patients with advanced disease.

The main aim of this critical systematic review of the literature is to assess the level of current knowledge on the molecular markers involved in the biology, behavior, and prognosis of TBSCC.

## 2. Results

### Retrieving Studies

**Figure 1:**
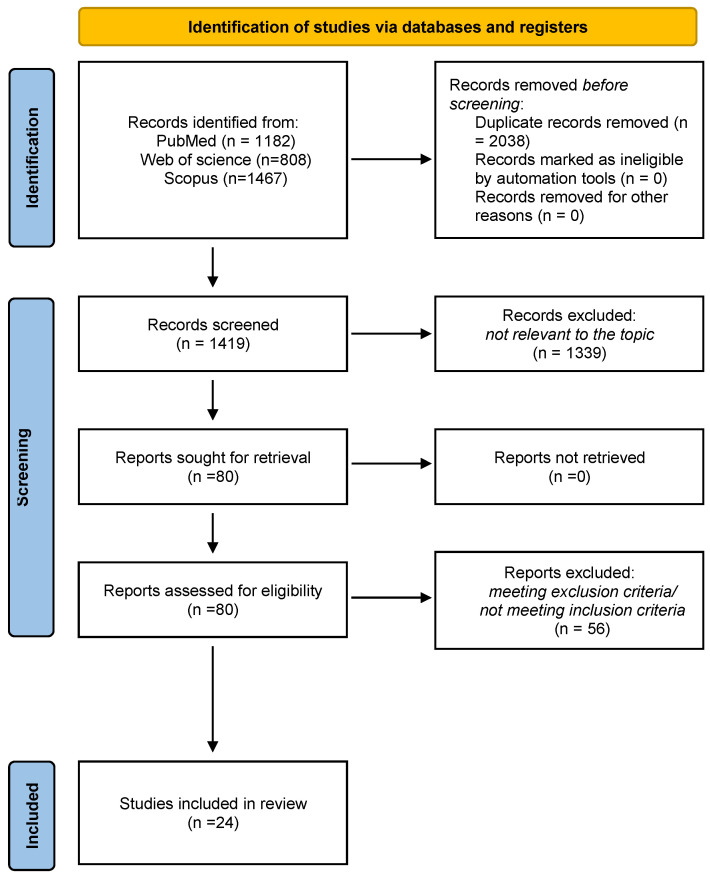
PRISMA flow diagram summarizing the literature review and inclusion/exclusion process.

A total of 3457 titles were retrieved (1182 from PubMed, 1467 from Scopus, and 808 from Web of Science). After removing duplicates, a total of 1419 titles were screened, allowing us to identify 80 studies potentially relevant to the topic. The full-text screening of these articles led to the exclusion of 56 studies due to their compliance with the inclusion/exclusion criteria. The remaining 24 articles were considered eligible for this review. A PRISMA flow diagram depicts the flow of information through the different literature review phases (Figure 1).

The studies included and analyzed in this systematic review have been summarized in Table 1.

## 3. Discussion

### 3.1. Human Papillomavirus Infection

Human papillomaviruses (HPVs) are a heterogeneous family of dsDNA viruses capable of infecting epithelial tissues. More than 100 different kinds of HPVs have been identified, some of which are involved in carcinogenesis. It has been demonstrated that HPV infection plays a causative role in some forms of head and neck SCCs. As regards the TBSCC subgroup, it has been found by polymerase chain reaction (PCR) that among 14 biopsies of SCC of the middle ear (MESCC), HPV16 alone was detected in six samples while HPV16 and HPV18 were simultaneously found in five [30]. None of the biopsies were positive for HPV-6, -11, -31, -33, 52b, or -58, which are some of the HPV types associated with head and neck cancers [32,33]. In situ hybridization showed a lower rate of positivity, while the cytopathic evidence was not effectively correlated with HPV presence in MESCC [30]. The higher sensitivity of PCR as compared to the other techniques supports its use for virus detection as a first choice. The high prevalence of oncogenic HPV types (79%) in MESCC suggests that HPV-16 and -18 are involved in cancer development. These data were confirmed in another study of nine biopsies of MESCC [31]. Tsai et al. [31] found an 88% prevalence of positive samples for the same HPV types, strengthening the hypothesis that HPV infection could be part of carcinogenesis. Expanding the inclusion criteria to both MESCC and EACSCC, Masterson et al. [26] detected HPV-16 in 3 out of 14 biopsies (22%) by PCR. The different detection rates may be due to the different races and ethnicities of the considered groups of patients. In addition, the validation of PCR results, for example, using DNA sequencing techniques, was not always performed, reducing the accuracy of HPV detection. In a very recent investigation [16], none of the 10 analyzed EACSCCs showed an increase in the CDKN2A (p16) protein, even those of the TP53 wild-type. These results highlight the need to not only clarify the clinical significance of HPV infection in TBSCC, but also to understand which technique is better for infection identification, and, subsequently, standardize it. Indeed, it has been previously demonstrated that PCR is more sensitive than immunohistochemistry in HPV detection in other SCC types [34].

### 3.2. Conventional and Recently Reported Histopathological Markers

Rare neoplasms occurring in regions with a complex anatomy, such as those of the ear and the temporal bone, deserve accurate and unified pathological reporting with standardized nomenclature and terminology, to allow appropriate patient management via a multidisciplinary approach, and multi-institutional intercountry data collection to guide cancer treatment and future research. The International Collaboration on Cancer Reporting (ICCR) provides guidelines for the reporting of biopsy and resection specimens of benign and malignant primary tumors of the EAC, middle ear, and inner ear [35]. The required conventional elements of the pathology reports are: (i) identification of the type of surgical resection performed, documentation of the various structures included, and the exact tumor site, as it correlates with outcome [36]; (ii) tumor size; (iii) histological tumor type and grading, according to WHO [37]; (iv) perineural and lymphovascular invasion; (v) extent of invasion; (vi) margin status; and (vii) pathological staging.

In the absence of a universally accepted staging system for TBSCC, pathologic T classification is frequently applied, which was founded on the revised Pittsburgh staging system [38], based on retrospective clinical and radiologic analysis of patients with EAC squamous cell carcinoma.

It is definitely recommended that in en bloc resections, the specimens should be sectioned in a manner that provides the best cross sections of the various anatomical structures and their relationship with the tumor, along with margin status, as such parameters supply critical prognostic information and guide adjuvant radiotherapy and/or chemotherapy [5,6,39].

Defining the typical routes of SCC spread in the temporal bone and anatomic barriers can help in planning surgery extent and anticipating any possible location of the disease not highlighted by preoperative imaging [39]. In their series of nine post-mortem examinations of advanced-stage TBSCC, Ungar et al. [10] found that, from the EAC, the tumor approached the middle ear via the tympanic membrane, whether intact or perforated, or by invading the mastoid air cells system through the posterior EAC wall. Tumor growth in the perilabyrinthine cells extended to the apex. Soft tissue barriers were identified in the vestibulo-stapedial ligament, which limited SCC invasion into the vestibule in two cases, and the round window membrane, which limited inner ear cavity penetration.

Tumor budding is defined as single cells or clusters of up to four cells, whereas poorly differentiated clusters (PDCs) are tumor cell clusters composed of five or more neoplastic cells. Tumor budding represents a very aggressive subpopulation of cancer cells, suggested to be the morphological expression of an epithelial–mesenchymal transition phenotype. A Japanese research team was the first to establish the adverse prognostic role of TBSCC in (i) high-grade tumor budding, associated with poorer prognosis regardless of disease stage in multivariate analysis [22], and (ii) high-grade PDCs, related to TNM stage and middle ear invasion, with treatment effect associated with poor progression-free survival as an independent parameter [18]. Tumor budding in TBSCC, regardless of its localization (the core of the tumor or invasive front) and irrespective of the risk grading system used, proved to be a reliable predictor of neck lymph node metastasis and poor prognosis in a series of 32 surgically treated TBSCC patients [4]. On the contrary, PDCs did not show any significant correlations with prognosis or clinicopathological variables examined.

### 3.3. Oncogenes/Tumor Suppressor Genes

Oncogenes and tumor suppressor genes link cell cycle control to tumor formation and development. Their control over cell division is lost upon genetic alterations, leading to their persistent activation or stable inactivation, respectively [40].

#### 3.3.1. Maspin

Human maspin (mammary serine protease inhibitor), identified originally in normal mammary epithelia, is encoded by SERPINB5, located on the long arm of chromosome 18 (18q21.3); it has a multitude of functions, which include modulating cancer cell motility and invasiveness, apoptosis, angiogenesis, and adhesion [41,42]. Maspin’s alternative tumor-suppressing/tumor-stimulating activity is related to epigenetic regulation, i.e., promoter methylation, which defines its subcellular localization (cytoplasm and/or nucleus) and varies among malignancies located in different organs/sites.

Marioni et al. [24] first investigated maspin expression in TBSCC by immunohistochemistry. They found higher maspin cytoplasmic staining in neoplastic cells of cases with low histological grade (G1/G2), and in those who did not develop disease recurrence. This supported the hypothesis of a promising prognostic role for maspin as an indicator of recurrence in TBSCC. Additionally, reactivating maspin functions by providing exogenous recombinant forms of the protein may be a potentially effective adjuvant treatment for advanced TBSCC, in combination with apoptosis-inducing and/or anti-angiogenic chemotherapeutic agents.

#### 3.3.2. p53, EGFR, and Notch1

Tumor suppressor p53 mainly exerts its role through the transcriptional regulation of its downstream target genes, forming a complex p53 signaling pathway that regulates a wide variety of biological processes to prevent tumorigenesis, including apoptosis, cell cycle arrest, and cellular senescence [43]. EGFR is a transmembrane glycoprotein expressed by the majority of epithelial tumors. Its activation leads to the initiation of intracellular signaling pathways, which starts proliferation, angiogenesis, invasion and metastasis [44]. The overexpression of EGFR, as well as p53, may be a predictive marker for the development of tumorigenesis in TBSCC. Considering a series of 30 TBSCCs, Morita et al. [19] discovered that both p53 and EGFR nuclear and membranous immunohistochemical expression in neoplastic cells was significantly associated with T classification, whereas EGFR expression alone was significantly more frequent in patients with lymph node metastasis compared to those without node involvement. The expression of EGFR and Notch1, a member of the NOTCH family of transmembrane proteins that regulate many basic processes essential for cancer development and progression [45], was significantly associated with poor survival outcomes in TBSCC. However, the low positivity rate (13.3%) of Notch1 in TBSCC, despite an absence of staining in normal tissue controls, seemed to limit its usefulness as a prognostic marker. A similar result, in terms of a negative prognostic role of EGFR, was found by [14] on a series of 22 TBSCC patients. In that series, five cases, accounting for recurrence on cervical nodes and locally advanced TBSCCs, were treated with cetuximab. In terms of a response to the targeted drug, EGFR overexpression was detected in one of the two patients that had remarkable cetuximab effects, as well as in two of the three cases that had no effect, suggesting no association between the effect of cetuximab and EGFR expression in a TBSCC cohort such as this.

#### 3.3.3. pSTAT3

In response to extracellular stimuli, the STAT family of transcription factors are phosphorylated, dimerize and translocate to the nucleus, where they target gene mediators of inflammation, cell survival, differentiation, and proliferation [46]. In more detail, several cytokines, such as interleukin-6 (IL-6) and interleukin-10 (IL-10), and growth factors comprising epidermal growth factor (EGF), initiate the activation of STAT3. The binding of these elements to their relevant receptors leads to the activation of Janus kinases (JAKs). The cognate receptor’s cytoplasmic tail is phosphorylated by JAKs, followed by the binding of the SH2 domain of STAT3 to phosphorylated tyrosine residues. The phosphorylation of STAT3 enables the translocation of signals from the cytoplasm to the nucleus by forming homodimers. Once translocated, pSTAT3 binds to the target genes’ promoter site by forming a complex with several coactivators, leading to the transcription of cyclin D1, survivin, and Bcl-xL [47,48].

Constitutively active phosphorylated-STAT3 (pSTAT3) acts as an oncogene, and has recently been described as a downstream effector of EGFR, interleukin-6, and Src family kinases in head and neck SCC [49]. In a series of 25 TBSCCs [25], neither carcinoma recurrence rate nor survival (DFS and DSS) were associated with pSTAT3 expression, maybe due to the limited number of cases examined and the single site of phosphorylation analyzed. In the series considered, normal stratified squamous epithelia showed diffuse nuclear immune labeling in both basal and spinous layers, scant immunoreaction in the granular layer, and none in the keratin layer, with most cases showing moderate-to-strong immunoreaction in a considerable proportion of carcinoma cells. Interestingly, pSTAT3 expression was stronger in areas of the tumor where cells were arranged in tiny strands or single cells, rather than in zones displaying large cohesive nests associated with keratin pearls, indicating an association with different TBSCC growth patterns. STAT3 downstream-activated genes, such as Cyclin D1, have not been proven to be related to TBSCC prognosis or other clinicopathological features [19]. On the contrary, a relevant role of EGFR in TBSCC has already been reported (see Section 3.3.2). Further investigations are therefore necessary to thoroughly clarify whether pSTAT3 has a relevant role in this tumor.

#### 3.3.4. KRAS

The oncogenic Kirsten rat sarcoma viral oncogene homolog (*KRAS*) represents one of the most frequently activated oncogenes in human carcinogenesis, with 17% to 25% of all human tumors displaying an activating *KRAS* mutation, typically affecting codons 12 and 13 in exon 2 [50]. In head and neck SCC, *KRAS* mutations have been investigated mainly in oral SCC and seem very infrequent [51], with no currently available data for TBSCC. The *KRAS*-variant was the first microRNA binding site mutation discovered in cancer, and is a functional germline mutation in the 3′ untranslated region. In head and neck SCC, the *KRAS*-variant, which is found in 15% to 32% of SCC cases tested, significantly benefits from the addition of cetuximab to radiotherapy and cisplatin [52].

### 3.4. Tumor Microenvironment in TBSCC

The microenvironment of a developing tumor is comprised of proliferating tumor cells, tumor stroma, an extracellular matrix (ECM), blood vessels, infiltrating inflammatory cells, and a variety of stromal-associated cells [53].

#### 3.4.1. ECM Degradation

The ECM and the basal membrane are the main barriers that can restrict cancer development by preventing tumor invasion and metastasis. A wide range of proteases, including matrix metalloproteinases (MMPs), have been implicated in the degradation of such barriers [54]. The reversion-inducing cysteine-rich protein with kazal motifs (RECK), initially considered a tumor suppressor gene, has been demonstrated to inhibit tumor invasion and metastasis by negatively regulating MMP2 and MMP9 [55]. The first attempt to elucidate these mechanisms in TBSCC was made by Liu et al. [28], who found significantly lower RECK expression in 30 MESCC samples than in normal external ear canal skin tissues, whereas the positive rate of MMP9 expression was higher in MESCC tissue than that in normal external ear canal skin tissue. The expressions of RECK and MMP9 were associated with tumor staging and grading, with a significant negative relationship between them. More recently, Aoki et al. [56] reported that extracellular matrix metalloproteinase inducer (emmprin) forms a complex with CD73, a cell surface protein regulating adenosinergic signaling and involved in tumor invasion processes. Subsequently, the same group [15] studied 34 pretreatment biopsy specimens of primary TBSCC, and reported an association between high tumoral emmprin expression and high-grade tumor budding, and between high stromal CD73 expression and high-grade PDCs. Concurrent elevated expression of tumoral emmprin and stromal CD73 was an independent poor prognostic factor for overall survival (OS) in multivariate analysis. Furthermore, high tumoral emmprin and stromal CD73 expression cases were significantly associated with middle cranial fossa invasion and disease recurrence. In vitro analyses confirmed the complex formation between emmprin and CD73, the more abundant production of MMP-2 coming from fibroblasts co-cultured with TBSCC cells than from those cultured alone, and the reduction in MMP-2 production by the transfection of CD73 siRNA into fibroblasts co-cultured with tumor cells [15].

#### 3.4.2. Basement Membrane Constituents and Cytoskeleton Remodeling

One of the major constituents of the basement membrane in most healthy tissues is laminin 5, a heterotrimer composed of three different laminin chains (α3-, β3-, γ2-), one of which, Ln5-γ2, is a specific marker for invasive tumors as it is frequently expressed as a monomer in several types of malignancies [57].

In their investigation on 46 preoperative biopsies of TBSCC cases, Okado et al. [22] stated that both tumor budding and Ln5-γ2 could be used as indicators of poor prognosis. High Ln5-γ2 cytoplasmic expression levels in carcinomatous cells were significantly associated with the presence of tumor budding and lower DSS. Moreover, high budding grade was significantly related to both a diffuse Ln5-γ2 expression pattern and high Ln5-γ2 expression levels.

As regards cytoskeleton remodeling in TBSCC, our group explored the role of cortactin and phosphorylated cortactin (residue tyr466) in 27 consecutively operated patients [20]. Cortactin is a multidomain protein, activated by tyrosine or serine/threonine phosphorylation, engaged in actin assembly and cytoskeletal arrangement. In the aforementioned series, despite the absence of a significant association between cortactin expression and recurrence rate and/or survival, this protein showed an upregulation in the cytoplasm of carcinoma cells, compared with normal adjacent tissue, supporting the hypothesis that inhibiting cortactin functions could have selective effects on this malignancy.

#### 3.4.3. The Immune Microenvironment: Programmed Death-Ligand 1 (PD-L1) and Other Checkpoint Inhibitors, Tumor-Infiltrating Lymphocytes (TILs), and Innate Lymphoid Cells

Despite the fact that PD-L1 is frequently expressed (up to 70%) in head and neck SCCs, its relation to prognosis is still debated. In selected recurrent or metastatic head and neck SCC cases, treatments with anti-programmed cell death-1 (PD-1) immune check-point inhibitors showed more favorable prognoses compared to treatment with conventional chemotherapies or cetuximab [58,59]. Nevertheless, TBSCC was not included in those clinical trials, nor has its immune microenvironment been sufficiently studied; however, Hongo et al. [1] analyzed a cohort of 123 TBSCC patients. In their series, the PD-L1 membrane expression of tumor cells was assessed in ≥100 tumor cells, and samples were defined as “positive” when membranous staining was detected in ≥1% of the tumor cells. PD-L1 detection rate was 50.4%, and was significantly associated with poor progression-free survival and OS. Low CD8+ TIL density and high Foxp3+ regulatory TIL density were each associated with unfavorable prognosis; a high CD8+ TIL density tended to be associated with a favorable response to chemoradiotherapy. In more detail, the so-called type III microenvironment (CD8+ low/PD-L1 +) was characterized by high T stage, high risk of nodal metastases, and poor prognosis.

Multiple immune cells coexist within the tumor microenvironment, including TILs (CD8+ T cells and regulatory T cells (Tregs)), NK cells, and macrophages. Head and neck SCCs are highly immune infiltrated, but are overall characterized by an immunosuppressive tumor microenvironment. Despite the heterogeneity of these studies and absence of investigations including TBSCC, increased infiltration by CD8+ T cells is the only immune cell type in head and neck SCC consistently proven to be correlated with increased survival regardless of tumor location, stage, and/or treatment [60]. Increased tumoral infiltration by CD8+ T cells, and an increased ratio of CD8+/T cells/Tregs, positively correlated with treatment response in a retrospective evaluation of 126 patients with advanced head and neck SCC treated with anti-PD-1 (programmed death-1)/PD-L1 agents [61].

In head and neck SCC, a high immunoscore (that is, the density of CD8+ T cells within the tumor center versus the invasive margin) has been associated with lower levels of Tregs, and increased PD-L1 and MHC type I expression in tumor cells [62], suggesting its potential to identify a subset of tumors with increased sensitivity to anti-PD-1/PD-L1 therapy. However, the predictive role of immunoscore in head and neck SCC has not been explored yet. The co-expression of other inhibitory immune-checkpoint molecules, such as TIM-3 (T-cell immunoglobulin and mucin domain-containing protein 3) and lymphocyte-activating gene 3 (LAG-3), has also been shown to impair immune T-cell-mediated responses, conferring resistance to anti-PD-1/PD-L1 agents in preclinical models and in patients across different tumor types. In head and neck SCC, a recent study showed that exhausted intratumoral PD-1+ CD8+ T cells expressing TIM-3 or LAG-3 were higher among non-responders to anti-PD-1 therapy [61].

NK cells are CD3-CD56+ innate lymphoid cells that possess features of both innate and adaptive immune cells, and are critical for the elimination of virally infected and transformed cells. Pan-cancer transcriptomic analyses have revealed that head and neck SCC are among the most highly infiltrated tumors, and exhibit the highest median CD56+ NK cell infiltration of any other tumor type. Increased infiltration of NK cells has been associated with improved DFS and OS, independent of HPV status [63].

#### 3.4.4. Epithelial–Mesenchymal Transition (EMT)

In the EMT process, epithelial cells lose polarity and cell-to-cell adhesions, undergo remodeling of the cytoskeleton, and activate the expression of mesenchymal components, thus gaining a migratory phenotype [64]. Considering in vitro studies, EMT seems to be induced, among other factors, by transforming growth factor-β1 (TGFβ1) [65]. Sugimoto et al. [29] hypothesized a link between EMT and bone invasion in TBSCC, as they found a significant increase in vimentin expression among patients with extensive bone involvement, and a co-localization of TGFβ-positive areas and neoplastic vimentin-positive cells, although exact overlap was not identified. This phenomenon implies that, in progressive TBSCC, TGFβ1, which is both released from destroyed bone and secreted from the tumor cells themselves, could induce EMT.

### 3.5. Neoangiogenesis

The early growth of solid tumors requires no neovascularization because tumors are no more than 2–3 mm in diameter; at this stage, the neoplastic tissue draws oxygen and nutrients, via diffusion, from adjacent vessels, and the tumor rarely metastasizes. When neoplastic cells begin to produce angiogenic factors, they recruit vessels from adjacent healthy tissues and start growing and developing metastases [66]. The “angiogenic switch” occurs when proangiogenic factors are not balanced by antiangiogenic factors: angiogenic autonomy is an essential step in the proliferation of neoplastic cells.

CD105 is a homodimeric transmembrane glycoprotein, and an auxiliary receptor of transforming growth factor-β (TGF-β) that binds to TGF-β1 and TGF-β3. It modulates TGF-β signaling by interacting with type I and type II TGF-β receptors, and activates a complex signaling pathway involving endothelial cell proliferation, migration, and adhesion [67]. The human CD105 gene is located on chromosome 9q34; the coding region contains 14 exons [68]. Furthermore, CD105 is active in angiogenesis [69]. A recent meta-analysis conducted on 30 studies involving 3613 cancer patients found strong evidence of increased CD105 expression in tumor microvessels, correlating with poor OS, DFS, and cancer-specific survival rates [70]. In 2012, Marioni et al. [27] investigated the prognostic role of CD105 expression in temporal bone SCC. CD105 immunohistochemical expression in intratumor microvessels endothelial cells was assessed by image analysis on 20 operated primary TBSCCs. The recurrence rate was significantly higher, and the DFS lower, in patients with CD105 expression of 9.44% or higher, than in cases where it was less than 9.44%. The crude carcinoma recurrence risk ratio was 5.9 times higher for TBSCC patients whose CD105 expression was 9.44% or higher. Marioni et al. [27] concluded that CD105 expression in activated endothelial cells of temporal bone SCC could be considered potentially useful for detecting patients at a higher risk of local disease recurrence after treatment.

Human relaxin-2 is a 6 kDa peptidic hormone characterized by a structure similar to that of insulin. Relaxin-2 has been associated with cancer biology [71]. A number of putative roles, including the modulation of tumor growth, neovascularization, metastasis, and oncogenic progression, have been correlated to its overexpression. Relaxin-2 has mostly been implicated in tumors that metastasize to bone, such as breast, prostate, and thyroid cancers, and myeloma, but it has also been associated with primary osteosarcoma [72]. A potential role for the relaxin-2 hormone in tumor-driven osteolysis has been reported [73]. In 2015, Marioni et al. [23] preliminarily investigated, in 25 consecutively operated TBSCC patients, the prognostic value of relaxin-2 expression in surgical specimen tissue and pathologically negative adjacent bone. The subcellular localization of relaxin-2 in the neoplastic cells was always cytoplasmic. Relaxin-2 stained some chondrocytes and osteocytes in the cartilage and bone tissue underlying and remote from the tumor, whereas there was no staining in either the cartilaginous or bone matrix. The recurrence rate, DFS, and disease-specific survival were not associated with relaxin-2 expression in either carcinoma specimens or in pathologically negative bone margins.

### 3.6. Systemic Inflammatory Markers

#### 3.6.1. White Blood Cell Counts

In several malignancies, the well-known relationship between systemic inflammation and tumor progression results in an association between circulating inflammation markers and prognosis [74,75]. In particular, circulating inflammatory blood cell counts and their derived indexes (i.e., neutrophil-to-lymphocyte ratio (NLR), platelet-to-lymphocyte ratio (PLR), and lymphocyte-to-monocyte ratio (MLR)) are gaining increasing interest as prognosticators for head and neck cancers, due to both their predictive value and wide availability [76,77,78].

In a Japanese series of 71 TBSCC cases treated with curative intent (radical surgery or chemoradiotherapy), Komune et al. [12] reported an absolute white blood cell count higher than 8300 cells/µL as a significant predictor of lower OS (HR, 3.244; 95% CI, 1.177–8.936).

Despite the fact that the comprehensive white blood cell count may reflect the overall systemic inflammation status, thus potentially predicting the outcome, each subpopulation of leucocytes may have a specific prognostic value [78,79]. As verified for other malignancies [78,79], in a Chinese cohort of 83 TBSCCs treated with primary surgery (temporal bone resection with parotidectomy), Li et al. [17] found that higher neutrophil counts seemed to predict poor outcome in terms of a higher recurrence risk. In the aforementioned cohort, the cut-off value calculated for outcome discrimination was 3.75 × 10^9^ cells/L. On the other hand, in the same series, increasing lymphocyte counts were found to be associated with a lower recurrence risk, with a suggested discrimination cut-off value of 1.77 × 109 cells/L [17].

#### 3.6.2. NLR, PLR, and LMR

Regarding the derived indexes, Makita et al. [11], in a series of 24 patients with locally advanced EACSCC or MESCC that received radiotherapy with or without surgery or systemic therapy as initial treatment, found higher NLR values to be associated with worse 1- and 2-year OS. A NLR value of 3.95 was suggested as a possible cut-off for prognostic discrimination. Komune et al. [12], in their series of 71 cases, found higher NLR values to be associated with reduced 5-year OS rates, also calculating, using an ROC approach, a NLR value of 3.23 as a prognostically relevant cut-off. They also found PLR values higher than 191.29 to be associated with lower OS. Accordingly, in their series of 83 surgically treated TBSCCs, Li et al. [17] also found NLR values higher than 2.325 to be predictors of recurrence, and to be associated with higher T stage at diagnosis [17]. In the same study, PLR values higher than 157.9 were found to be associated with higher recurrence risk.

Although literature focusing on this topic is based on only a few articles [11,12,17], and, consequently, on a limited number of cases, the existing evidence seems to be remarkably concordant in depicting a higher NLR value as a negative prognostic factor. Conversely, increasing LMR values emerged as a promising positive prognostic factor: values higher than 3.78 were found to predict a higher OS [12], whereas values higher than 3.065 were associated with lower recurrence rate [17].

#### 3.6.3. Blood C-Reactive Protein (CRP)

The C-reactive protein (CRP) is produced by hepatocytes as a systemic response to cytokine stimulation (particularly interleukin-6) from tumor microenvironment leukocytes, and has been associated with progressive disease and relatively poor survival in patients with different malignancies [80,81]. Regarding the specific setting of TBSCC, CRP values higher than 1 mg/dL were associated with lower OS [12].

Inflammation is often associated with decreased serum albumin levels, owing to suppressed liver function [82]. This concept led to the proposal of the CRP-to-albumin ratio (CAR) as a tool for assessing the extent of inflammation in a tumor microenvironment and, consequently, predicting prognosis [83,84]. In TBSCCs, Makita et al. [11] found significantly reduced DFS and OS in patients with a pretreatment CAR value ≥0.31, further supporting the concept that high systemic inflammation markers at diagnosis are also associated with a dismal prognosis in this specific tumor.

### 3.7. Genetic Landscape

Genetic analyses in this type of cancer are applied in pioneering studies, and are thus limited by the size of the sample; the aims of these studies are to highlight associations useful for the diagnosis and/or prognosis of the tumor, rather than to optimize new approaches in precision medicine. For diagnostic purposes, there are some investigations in which molecular screening for the presence or absence of HPV and EBV is carried out to validate its correlation with carcinogenesis [26,30,31]. Among the first studies in which whole exon sequencing (WES) was performed, a case report on a patient with recurrent TBSCC cannot be omitted [85]. The results obtained showed the presence of mutations, albeit missense mutations, in the genes β-catenin 1 (CTNNB1) and vascular endothelial growth factor receptor 2 (VEGFR-2). β-catenin is involved in cell adhesion and gene transcription, and alterations in its expression have already been reported as possible prognostic biomarkers in oral squamous cell carcinoma [86]. As an angiogenetic promotor, VEGF is one of the target genes of precision medicine [87], which is why, after verifying the overexpression of VEGFR-2 and VEGF, the patient was treated with Bevacizumab, an anti-VEGF monoclonal antibody, in combination with pemetrexed disodium, a folate analogue [85]. The reported 95% carcinoma reduction supports the intriguing role of targeted therapy on molecular bases as a treatment option, especially in cases of recurrent cancer [85]. Another molecular marker that has been considered for this purpose is DEK–AFF2 fusion; this fusion is characteristic of sinonasal tumors, but has been identified in some cases even in the ear/temporal bone. Unfortunately, the small number of cases examined did not allow any significant clinical interpretations [88,89].

In more recent studies, whole exome sequencing on primary EACSCC has been independently performed, from which it has been confirmed that the most frequently mutated gene is TP53, followed by thyrosin kinase receptor (e.g., EGFR, FGDR) genes involved on P13k, Notch, and FAT1 pathways, as well as amplifications of the chromosomal regions of 3q, 5p, and 8q [13,16]. All these genes have already been described in the oncogenesis of many tumors, including head and neck SCC; in fact, they are part of a panel of genes that is commonly investigated in this setting. This panel has been also considered useful to establish and characterize primary cell cultures derived from EACSCC [13,16,90]. Among the modified genes which could be considered peculiar to the EACSCC, rather than head and neck SCC, the telomerase reverse transcriptase (TERT), DNA methyltransferase 1 (DNMT1) [13], the encoding finger type DHHHC-containing 11B (ZDHHC11B), and the T-cell receptor gamma chain (TARP) [16]. TERT and DNMT1 lead to chromosomal and chromatin instability, resulting in epigenetic dysregulation typical of carcinogenesis [91,92]. ZDHHC11B has been described as an oncogene that promotes cell proliferation, while TARP also promotes tumor cell invasion [93,94]. Quite recently, by a transcriptome investigation, a new possible prognostic biomarker was identified for EACSCC, the long non-coding RNA MMP3-1. It is a lncRNA of unknown biological function whose expression in EACSCC has been preliminarily associated with limited tumor invasion and longer survival [21]. Liu et al. [21] highlighted the importance of MMP3-1 as a possible biomarker, although it needs further analysis from the functional point of view and, above all, an increase in the number of cases investigated.

## 4. Materials and Methods

A search was run in the PubMed [95], Scopus [96], and Web of Science [97] electronic databases for papers (without publication date limits) that investigated molecular markers in cohorts of patients with primary TBSCC.

The search terms used were: “temporal bone” OR “external auditory canal” OR “ear”, AND “cancer” OR “carcinoma” OR “malignancy”.

The “Related articles” option on the PubMed homepage was also considered. We examined the titles and abstracts of papers available in the English language. The full texts of the publications identified were screened for original data, and the references in the articles retrieved were checked manually for other relevant studies.

The literature search has been updated to 21 March 2022.

### 4.1. Inclusion/Exclusion Criteria

Studies were included when the following general criteria were met: (i) articles were an original report; (ii) the study design was not a case report, editorial, letter to the editor, or review; (iii) the histology of the reported cases was squamous cell carcinoma; (iv) a biological marker was investigated in relation to TBSCC features and/or prognosis; (v) the report was published in the English language; (vi) the study population contained at least 5 patients.

### 4.2. Data Extraction

Two of the authors (L.F., G.M.) extracted the data from the selected articles. Disagreements were dealt with by discussion among the team members. In the case of studies also reporting information on histological entities other than squamous cell carcinoma, only data about patients with this specific histotype were considered for this review’s purpose.

## 5. Conclusions

Given the poor prognosis for patients with advanced primary TBSCC, it is of the utmost importance to identify patients at higher risk of recurrence in order to plan integrated treatment approaches, including surgery with neoadjuvant or adjuvant therapy (chemo- or radiotherapy, respectively). Early recognition of patients with a poor prognosis through clinical and radiological evidence, and also predictive biomarkers, would be crucial for choosing aggressive therapy (extended surgery). On the other hand, appropriate biomarkers could play a role in identifying those cases in which prognosis is so poor that palliation could be the only rational approach. These considerations corroborate the clinical need for novel prognostic markers.

In the present systematic review of the limited available data on molecular markers and TBSCC (see also Table 1), it was found that the investigations were “case series” (observational studies without controls), achieving a level of evidence 4, according to the Oxford Centre for Evidence-based Medicine: Levels of Evidence [98]. Although only very limited information on the prognostic role of molecular markers in TBSCC is currently available, some findings are definitely promising.

Prospective, multi-institutional prognostic studies—preferably on an international scale—should be planned to identify the molecular markers involved in the biology, clinical behavior, and prognosis of TBSCC, concomitantly analyzing their potential clinical application. Biomarker identification could rely on many different approaches, such as fluorescence in situ hybridization, immunohistochemistry, and omic sciences (genomics, transcriptomics, proteomics, metabolomics, and lipidomics) [99]. For the candidate biomarkers discussed in this review, it seems reasonable to focus on gene and protein evaluations based on PCR/sequencing and immunohistochemistry, respectively. The application of next generation sequencing, including exome sequencing, as well as multispectral tissue immunohistochemistry, seems to be an attractive course of action to further broaden the horizon of biomarker discovery.

## Figures and Tables

**Table 1:** Association between biomarker expression, main clinical and pathological variables, and prognosis in TBSCC.

Authors/Year	Potential Biomarker Considered	Methods	No. of Cases	Follow-Up Period	T *	N	Stage	G	Recurrence Rate	Survival	Remarks
Alessandrini et al., 2022 [4]	PTB ITB PDC	*Conventional histopathology*	32	71 (10–23) months	PTB - ITB + PDC -	PTB + ITB - PDC -	ND	PTB - ITB - PDC -	PTB + ITB - PDC + (trend)	PTB (DFS +, OS +)	ITB and PTB evaluated as either absolute counts or 2-tier and 3-tier scores.
Ungar et al., 2021 [10]	Invasion pattern	*Conventional histopathology* *Macrosections*	9	ND	ND	ND	ND	ND	ND	ND	Retrospective analysis of medical charts and ex vivo temporal bone specimens of patients diagnosed with TBSCC.
Makita et al., 2021 [11]	NLR CAR PLR AGR	*Blood tests*	24	25 (6–137) months	ND	ND	ND	ND	NLR - CAR + PLR - AGR -	NLR (OS +, DFS -) CAR (OS +, DFS +) PLR (OS -, DFS -) AGR (OS -, DFS -)	All included patients were T3–4.
Hongo et al., 2021 [1]	PDL1 Foxp3 CD8	*IHC*	123	ND	-	-	-	-	PDL1 + Foxp3 + CD8 +	PDL1 (OS +, DFS +) Foxp3 (OS +, DFS +) CD8 (OS +, DFS +)	Retrospective analysis of collected biopsied or surgically resected specimens from 123 TBSCC cases.PD-L1 and Foxp3 expression were significantly associated with worse prognosis. A high density of CD8+ TILs was significantly associated with better prognosis.
Komune et al., 2021 [12]	WBC PLT LMR NLR PLR	*Blood cell count*	71	ND	LMR - NLR - PLR +	LMR - NLR + PLR +	ND	ND	ND	LMR (OS +) NLR (OS +) PLR (OS +)	Inflammation-based prognostic markers were associated with the survival.
Basura et al., 2021 [13]	TP53 EGFR FGFR PI3K	*Copy number analysis*	7	ND	ND	ND	ND	ND	ND	ND	Targeted DNA sequencing in 7 TBSCCs using a 227-gene panel.
Maki et al., 2021 [14]	p53 EGFR	*IHC*	22	63 (5–132) months	p53 - EGFR -	ND	ND	p53 - EGFR -	ND	p53 (OS -) EGFR (OS +)	-
Miyazaki et al., 2020 [15]	CD73 TB PDC t-emmprin	*IHC* *Immunoblotting*	34	ND	ND	ND	ND	ND	ND	CD73 (OS -) TB (OS +) PDC (OS +) t-emmprin (OS +) CD73 and t-emmprin (OS +)	High-grade TB and PDCs are associated with shorter survival. Concurrent elevated expression of t-emmprin and stromal CD73 was a poor prognostic factor.
Sato et al., 2020 [16]	TP53 CDKN2A NOTCH1 NOTCH2 FAT1 FAT3	*WES*	10	ND	ND	ND	ND	ND	ND	ND	WES performed on 11 primary tumors, 1 relapsed tumor, and 10 noncancerous tissues from 10 patients with TBSCC.
Li et al., 2020 [17]	NEU MON LYM PLT LMR NLR PLR	*Blood cell count*	83	27(8–138) months	NEU + MON - LYM - PLT - LMR - NLR + PLR -	ND	ND	-	NEU + LYM + LMR + NLR + PLR +	ND	Preoperative neutrophil and lymphocyte counts, NLR, PLR, and LMR were significantly correlated with tumor recurrence.
Miyazaki et al., 2019 [18]	PDC TB	*Conventional histopathology* *IHC*	31	3 years	PDC +	PDC -	PDC +	PDC +	PDC + (trend)	PDC (PFS +) TB (PFS + [trend])	In multivariate analysis, high-grade PDCs were associated with poor prognosis.
Morita et al., 2018 [19]	p53 p16 cyclin D1 EGFR Notch1	*IHC*	30	9−112 months	p53 + p16 - cyclin D1 - EGFR + Notch1 -	p53 + p16 - cyclin D1 - EGFR + Notch1 -	ND	p53 - p16 - cyclin D1 - EGFR - Notch1 -	ND	p53 (OS -) p16 (OS -) cyclin D1 (OS -) EGFR (OS +) Notch1 (OS +)	EGFR and Notch1 were significantly correlated with poor survival outcomes.
Marioni et al., 2017 [20]	Cortactin	*IHC*	27	82.9 ± 67.1 months	-	-	-	-	-	DFS -	Cortactin expression was higher in carcinoma cells than in normal tissue. Recurrence and DFS rates did not correlate with cortactin expression.
Liu et al., 2017 [21]	lncRNA MMP 3-1	*ISH*	8	3 years	+	-	+	+	ND	OS +	Lnc-MMP3-1 was the most upregulated lncRNA in EACSCC, with fold change of 237.2.
Okado et al., 2015 [22]	laminin5-γ2 TB	*IHC*	46	34.6 (4–66) months	laminin5-γ2-TB	laminin5-γ2-TB	laminin5-γ2-TB	laminin5-γ2-TB	laminin5-γ2-TB + (trend)	laminin5-γ2 (DSS +) TB (DSS +)	Multivariate analysis revealed that high budding grade predicted poorer prognosis regardless of disease stage.
Marioni et al., 2015 [23]	Relaxin-2	*IHC*	25	76.0 months (median)	-	-	ND	-	-	DFS - DSS -	-
Marioni et al., 2013 [24]	MASPIN	*IHC*	29	50.0 months (median)	-	-	ND	+	+	DFS - DSS -	MASPIN in the cytoplasm.
Marioni et al., 2013 [25]	pSTAT3	*IHC*	25	42.0 months (median)	-	-	-	-	-	DFS - DSS -	-
	HPV	*ISH*, PCR	14	ND	ND	ND	ND	ND	ND	-	Nested PCR was positive in 3 of 14 cases: DNA sequencing of positive samples revealed the HPV16 subtype in all cases. HPV-associated patients showed a trend towards improved survival.
Masterson et al., 2013 [26]	EBV	*ISH*, PCR	20	34.0 months (mean)	ND	ND	ND	ND	ND	ND	Out of 20 cases, 1 showed evidence of EBV positivity on PCR.
	p16	*IHC*	14	ND	ND	ND	ND	ND	ND	ND	Out of 3 HPV+ cases, 2 showed p16 staining; controls negative for HPV-DNA showed no evidence of p16 activity.
	TP53 mutation	PCR	13	ND	ND	ND	ND	ND	ND	ND	A functional mutation was found in 3 of 10 HPV- and in 0 of 3 HPV+ samples.
Marioni et al., 2012 [27]	Endoglin (CD105)	*IHC*	20	40.6 months (mean)	-	-	-	-	+	DFS +	-
Liu et al., 2012 [28]	RECK MMP9	*IHC*	30	ND	+ **	-	ND	ND	ND	ND	All middle ear SCCs. RECK and MMP9 expressions were higher for histological grades I–II than for grades III–IV.
Sugimoto et al., 2011 [29]	Vimentin TGF β	*IHC*	16	34.2 months (mean)	ND	ND	ND	ND	ND	Vimentin (DSS -)	Increase in score for vimentin expression in patients with extensive bone involvement.
Jin et al., 1997 [30]	HPV types 16 and 18	*ISH*, PCR	14	ND	ND	ND	ND	ND	ND	ND	All middle ear SCCs in patients with history of chronic otitis media: 5 cases were HPV16- and HPV18-positive; 6 cases were only HPV16-positive.
Tsai et al., 1997 [31]	HPV types 16 and 18	PCR	9	ND	ND	ND	ND	ND	ND	ND	Total of 8 middle ear SCCs and 1 adenocarcinoma: 8 patients were HPV+; 3 cases were HPV16- and HPV18-positive; only 4 SCCs and 1 adenocarcinoma were HPV16-positive.

Abbreviations: AGR, albumin-to-globulin ratio; CAR, CRP-to-albumin ratio; DSF, disease-free survival; DSS, disease-specific survival; EMT, epithelial–mesenchymal transition; *IHC*, immunohistochemistry; *ISH*, in situ hybridization; ITB, intratumor budding; LMR, lymphocyte-to-monocyte ratio; LYM, lymphocytes; MON, monocytes; ND, no data; NEU, neutrophils; NLR, neutrophil-to-lymphocyte ratio; PCR, polymerase chain reaction; PDC, poorly differentiated clusters; PFS, progression-free survival; PLR, platelet-to-lymphocyte ratio; PLT, platelets; PTB, peritumor budding; OS, overall survival; TB, tumor budding; WES, whole exome sequencing; +, significantly associated; -, not associated; *, revised Pittsburgh classification; **, Stell and McCormick staging system.

## Data Availability

The data presented in this study are available on request from the corresponding author.
